# A Malaysian version of learning space preferences: a validation study

**DOI:** 10.5116/ijme.6082.7c41

**Published:** 2021-05-27

**Authors:** Joong Hiong Sim, Chan Choong Foong, Vinod Pallath, Wei-Han Hong, Jamuna Vadivelu

**Affiliations:** 1 Medical Education and Research Development Unit, Faculty of Medicine, University of Malaya, Kuala Lumpur, Malaysia

**Keywords:** Validation, learning space questionnaire, learning space preferences, Malaysian medical school

## Abstract

**Objectives:**

This study aimed to validate a Malaysian version of a revised learning space questionnaire, as well as to test the utility of the revised questionnaire as a tool to investigate learning space preferences in a Malaysian medical school.

**Methods:**

This is a cross-sectional survey. A convenient sample of 310 preclinical students of a public medical school in Malaysia were invited to participate. Validation data were collected using a revised 40-item, 5-point Likert scale learning space questionnaire.  The questionnaires were administered online via a student e-learning platform.  Data analysis was conducted using IBM SPSS version 24.  Exploratory factor analysis was conducted to examine the factor structure of the revised questionnaire to provide evidence for construct validity.  To assess the internal consistency of the revised questionnaire, Cronbach's alpha coefficients (α) were computed across all the items as well as for items within each of the factor.

**Results:**

A total of 223 (71.94%) preclinical students completed and returned the questionnaire. In the final analysis, exploratory factor analysis with principal axis factoring and an oblimin rotation identified a six-factor, 20-item factor solution. Reliability analysis reported good internal consistency for the revised questionnaire, with an overall Cronbach's alpha of 0.845, and Cronbach's alpha ranging from 0.800 to 0.925 for the six factors.

**Conclusions:**

This study established evidence for the construct validity and internal consistency of the revised questionnaire.  The revised questionnaire appears to have utility as an instrument to investigate learning space preferences in Malaysian medical schools.

## Introduction

Learning space refers to a setting for a learning environment, a place in which teaching and learning occur,^1^ but it may also refer to an indoor or outdoor location, either physical or virtual.  The use and design of space in higher education is a theme that has come to the forefront of educators' interest in the past several years. It has long been accepted that space quality and design impacts the educational experience.^1^^-^^5^

The physical and/or virtual characteristics of learning spaces play an important role in their effectiveness and, by impacting students learning, on society. Beckers and colleagues^6^ found students consider their physical learning space to be relevant and suggested that learning spaces contribute to the outcome of their study activities.  Students mainly prefer learning spaces related to their learning activities.  There are individual differences among students resulting in different preferences and needs.^7^ Information and communication technology (ICT) are transforming the way learning spaces are used and configured.^8^ Its robust tools support the creativity of thought and allow learning to occur almost everywhere. In the past two decades, various changes occurred in the higher education system.  New learning objectives, the increased use of ICT facilities in education, and changed instructional methods are indicated as new ways of learning.^9^ New ways of learning are expected to require changes in the physical environment.^10^ Webb and colleagues^11^ concluded that "there is a growing awareness that learning happens all over the campus, not just in classrooms and labs".  Modern ICT facilities support new learning methods and allow students to study anytime, anyhow, and anywhere.  For example, flipped classroom concepts combine class attendance with e-learning at home or anywhere else. In these concepts, the main reason to visit a building for higher education is to meet other students and work collaboratively with tutors and peers. It is expected that higher education institutions have to offer their students more of these alternative learning spaces.^12^ Understanding of learning spaces would help educators reconsider their learning spaces' design to meet learners' requirements.

Globally, there are numerous studies on learning spaces in higher education practice.^3^^,^^13^^-^^17^ However, some of these studies^13^^,^^14^^,^^18^ only focused on an informal setting for learning, while some studies are qualitative in nature. Ibrahim and colleagues^14^ conducted a case study on learning outside the classroom on campus ground in a Malaysian public university. Furthermore, relatively few learning spaces study instruments have been psychometrically evaluated, while some of these instruments are context-specific. For example, Yan and Huan^2^ developed and validated a scale for evaluating technology-rich classroom, while MacLeod and colleagues^19^ developed and validated an instrument to measure student preferences towards the smart classroom.  In Malaysia, there is still a lack of empirical research on learning space preferences in higher education. There are 32 medical schools in Malaysia. However, to the best of our knowledge, there is no documented study on learning space preferences in Malaysian medical schools using a psychometrically validated questionnaire. Beckers and colleagues^6^ surveyed the learning space preferences of higher education students involving 697 business management students of a Dutch University of Applied Sciences while we intended to conduct our study on learning space preferences in a medical school in Malaysia.  Due to cultural and contextual differences, the original questionnaire used by Beckers and colleagues must be revised to adapt to our study setting. This study aimed to establish the construct validity and internal consistency of a revised learning space questionnaire (LSQ) and to test the utility of the revised LSQ as a tool to investigate learning space preferences in Malaysian medical schools.

## Methods

### Study design

As part of the validation process and to test the utility of the revised instrument, a cross-sectional survey was conducted.

### Sampling and recruitment of participants

A convenient sample of preclinical students from the Faculty of Medicine of a public university in Malaysia was invited to participate in this study. All the 310 preclinical students in Year 1 and Year 2 who consented to participate in the study were included.  This study was approved by the University Research Ethics Committee of our institution.  Informed consent was provided by all respondents.  Data collected were kept confidential and responses were anonymous.

### Study instrument

A revised 40-item, 5-point Likert scale questionnaire on learning spaces preferences adopted and adapted from Beckers and colleagues^6^ was used to collect data for this study.  Permission to use the questionnaire had been granted by the first author via personal communications on Research Gate.  The original questionnaire^6^ comprised of 38 items in five dimensions, namely: (i) Relevance of learning space (3 items), (ii) social dimension of the learning space (7 items), (iii) Physical dimension of the learning space (12 items), (iv) Learning space preferences for individual study activities ((8 items), and (v) Learning space preferences for collaborative study activities (8 items).

For "relevance of learning space" and "social dimension of the learning space" respondents were asked to indicate their preferences for each item based on a 5-point Likert scale from 1=I fully disagree to 5=I fully agree.  For "physical dimension of the learning space", respondents indicated their preferences for each item from 1=Very Unimportant to 5=Very Important.  For "learning space preferences" for both individual and collaborative study activities, respondents were asked to indicate their preferences for several prescribed learning spaces from 1=Absolutely not preferred to 5=Definitely preferred. 

### Revising the questionnaire

The revised questionnaire retained the five dimensions, but the number of items in the physical dimension was increased from 12 items to 14 items, with two items removed and four items added.  The two items removed from the original questionnaire were: "The finish of the floors in the building" and "A central location of learning settings in the building".^6^ These two items were removed as the contents of the items were irrelevant to our study setting.  Instead, four items (Items 20, 21, 22, 23) relevant to our study setting were added.  The five dimensions in the revised instrument are "relevance of learning space" (Items 1 to 3), "social dimension of learning space" (Items 4 to 10), "physical dimension of learning space" (Items 11 to 24), "learning space preferences for individual study activities" (Items 25 to 32) and "learning space preferences for collaborative study activities" (Items 33 to 40).

### Content validity

In this study, the physical dimension of the learning space was operationalised in four characteristics: the perceived importance of comfort, aesthetics, ICT facilities, and layout, while the degree of interaction, privacy, and Autonomy are used to operationalise the social dimension.  The revised learning space questionnaire (LSQ) was given to four medical educationists for review.  All four reviewers came to a consensus that the revised LSQ appeared to have face validity and content validity. 

### Study setting

The medical programme in our institution is a 5-year programme divided into preclinical (two years) and clinical (three years).  It is a multidisciplinary integrated programme with early clinical exposure beginning the first year.  In our revised medical curriculum, there is a combination of both individual and collaborative learning activities. Learning spaces are both physical and virtual.  Blended learning is facilitated through activities on an e-learning platform called SPeCTRUM.  As Nordquist and colleagues^20^ suggest, analysis of the changing curriculum should be accompanied by an evaluation of the set of appropriate learning spaces and to identify the spaces that may be needed in the future.     

### Data collection

The administration of questionnaires was conducted online via a student e-learning platform called SPeCTRUM.  Students were notified via the student bulletin board in SPeCTRUM to respond to the questionnaire.  However, participation was on a voluntary basis.  Data collection commenced in December 2018 and ended in January 2019.  One month after the commencement of data collection, about 50% of the students had responded to the questionnaire.  Upon sending a reminder to students, the response rate increased to about 72%.

### Data analysis

Analyses of items in the questionnaire were conducted using IBM SPSS Statistics version 24.  Both descriptive and inferential statistics were employed. 

#### Factor analysis

To examine the construct validity of the revised instrument, the factor structure of the revised LSQ was examined through exploratory factor analysis (EFA). EFA provides the tools for analysing the structure of the interrelationships (correlations) among a large number of variables/items by defining sets of variables/items that are highly interrelated, known as factors. These groups of variables (factors) are assumed to represent dimensions within the data.^21^ This statistical procedure is usually conducted during the development and validation of a new measure or when adapting a measure to a new population to produce evidence for the construct validity of the measure.^22^ EFA was used as we intended to explore the factors related to learning space as represented by a set of variables/items that describe the learning spaces, and subsequently, to summarise the structure of the set of variables/items.^23^

To assess the suitability of the respondent data for EFA, Kaiser-Meyer-Olkin (KMO) measure of sampling adequacy^24^ and Barlett's Test of sphericity^25^ were conducted. The KMO index ranges from 0 to 1, with 0.50 considered suitable for factor analysis.^21^^,^^26^ A statistically significant Barlett's Test of sphericity indicates that sufficient correlations exist among the variables to proceed with EFA.

In this study, principal axis factoring was selected as the factor extraction method. Principal axis factoring (PAF), also known as common factor analysis, is used primarily to identify underlying factors/dimensions that reflect what the variables share in common.^21^ PAF is most appropriate when the primary objective is to identify the latent dimension/constructs represented in the common variance of the original variables/items.^21^^, ^^22^

According to Hair and colleagues,^21^ any decision on the number of factors to be retained should be based on several considerations, including Kaiser's criteria or eigenvalue greater than 1.0 rule,^27^ the Scree test^28^ and the cumulative percent of variance extracted. For the first attempt at interpretation, a criterion such as the eigenvalue could be used as a guideline. This criterion is most reliable when the number of variables is between 20 and 50, and communalities above 0.40.^21^ The Scree test is used to identify the optimum number of factors that can be extracted, i.e. factors before the inflexion point.^28^ However, interpreting the Scree plot could be subjective, requiring researcher judgement.^26^ In this study, an initial minimum eigenvalue of 1 was used as the criteria to identify factors to retain.^29^ Graphically, Scree plots were examined with the eigenvalue restriction and based on the natural breakpoint of the data for the curve flattening out.^24^^, ^^28^ In addition, for practical significance for the derived factors, ensure that they explained at least a specified amount of variance, usually 60% or higher.^21^

To interpret the factors, we evaluated the initial results of EFA, then made a number of judgements in viewing and refining these results. Several iterations are necessary before a final solution is achieved.^21^ The steps involved in interpreting a factor matrix are: (i) examine the factor matrix of loadings, (ii) identify the significant loadings for each variable, (iii) assess the communalities of the variables, and (iv) respecify the factor model if needed. In interpreting the factors, we were guided by some general principles suggested by Hair colleagues^21^ such as: (i) an optimal factor structure exists when all variables have high loadings on only one factor, and very low loadings on all other factors, (ii) cross-loadings of a variable can be evaluated by the ratio of their squared loadings and classified as problematic (ratio between 1.0 and 1.5), potential (ratio between 1.5 and 2.0) or ignorable (ratio greater than 2.0), problematic and perhaps potential cross-loadings are deleted unless theoretically justified, (iii) variables should generally have communalities of greater than 0.50 to be retained in the analysis, and (iv) respecification of EFA results can include such options as deleting a variable(s), changing rotation methods, and/or increase or decrease the number of factors.

#### Reliability analysis

To assess the internal consistency of the revised LSQ and its factors, Cronbach's alpha coefficients (α) were computed across all the items as well as for items within each of the factor for all the completed questionnaires.

#### Descriptive statistics

To test the utility of the revised LSQ as a study instrument to investigate learning space preferences among medical students, mean score and standard deviation for each item as well as for each of the factor were computed. 

## Results

Of the 310 invited participants, 223 (71.94%) completed and returned the questionnaires. Data of the 223 preclinical students were included in the analysis.

### Factor analysis

Bartlett's test of sphericity, which tests the overall significance of all the correlations within the correlation matrix, was significant (χ^2^(780) = 4762.442, p<0.001), indicating that it was appropriate to use the factor analytic model on this set of data. The Kaiser-Meyer-Olkin measure of sampling adequacy indicated that the strength of the relationships among variables was high (KMO =0.807); thus it was acceptable to proceed with the analysis.  Preliminary analysis of the 40 items using exploratory factor analysis (EFA) with principal axis factoring (PAF) and varimax rotation identified 11 factors with eigenvalues over Kaiser's criterion of 1, which together accounted for 59.76% of the variance.  By Kaiser's criterion, we should extract 11 factors. However, the Scree plot showed no clear inflexion point. Results of the rotated component matrix also suggested that Items 29, 32, 33, 34 and 40 should be removed due to problematic cross-loadings. Although Items 26, 27, 28, 30, 35, 36 and 38 were extracted into the same factor, which was logical as they all appeared to be learning spaces that were open and busy, Item 25 and Item 33 were extracted into different factors although both referred to learning space at home.  A further check revealed similar problems with Item 31 and Item 39. In summary, these items were extracted into factors that did not appear to be relevant or meaningful factors/constructs. Communalities of items 25 to 40 revealed 9 out of the 16 items had communality of less than 0.5 (ranging from 0.308 to 0.495). An attempt to conduct an EFA separately for Items 25 to 40 also failed to extract any meaningful factors/constructs. A decision was made to exclude the 16 items (Items 25 to 40) from factor analysis and rerun EFA with PAF and varimax rotation for only the first 24 items.

Seven factors with eigenvalues greater than 1 were retained, which explained 63.74% of the variance.  KMO recorded a value of 0.816, with Bartlett's test highly significant (χ^2^(276) = 3005.444, p<0.001).  Items 15, 16, 17 and 24 were extracted into the same factor. While items 15, 16 and 17 recorded high factor loadings of 0.743, 0.866 and 0.751, respectively, item 24 only had a factor loading of 0.435. Communality for Item 24 was 0.262, which was undesirable. Hence, respecification of the model was necessary, and another attempt was made to rerun EFA without Item 24.

EFA with PAF and varimax rotation on the 23 items yielded seven factors with eigenvalues greater than 1, which together explained 65.41% of the variance. KMO recorded a value of 0.811, with Bartlett's test highly significant (χ^2^(253) = 2934.875, p<0.001). However, the initial eigenvalue revealed that the last factor extracted (F7) had an eigenvalue of 1.018, just above the minimum acceptable value of 1. Examination of the Scree plot showed no clear inflexion point. Two of the items (Items 1 and 2) which were extracted into F6 were found to have a communality of 0.415 and 0.463, respectively. Since F6 contained three items, removing Items, 1 and 2 would leave only one item. Hence, together with Item 3, all three items in F6 were removed and EFA based on PAF was attempted on the remaining 20 items.

EFA with PAF and varimax rotation extracted six factors with eigenvalues greater than 1, which together explained 67.19% of the variance, an increase of 7.43% compared to the 11-factor model in the preliminary analysis. KMO recorded a value of 0.800, with Bartlett's test highly significant (χ^2^(190) = 2644.614, p<0.001).  The rotated factor matrix showed three items (Items 18, 21 and 22) loaded significantly onto two factors. Cross-factor loadings for Item 18, Item 21 and Item 22 were (0.508, 0.507), (0.545, 0.647), and (0.499, 0.462) respectively. Cross-loadings for all these three items were problematic (with the ratio of the squares of the larger loading to the smaller loading less than 1.5). These cross-loadings could be due to correlations between some of the factors. Hence, another attempt was made to respecify the existing model by changing the rotation method.

For EFA with PAF and oblimin rotation on the 20 items, the pattern matrix showed Item 18, Items 21 and Item 22 still loaded on two factors. However, only the cross-loading for Item 22 was problematic. Cross-loading for Item 18 was designated as potential cross-loading, while cross-loading for Item 21 was ignorable. For ignorable cross-loadings, the smaller loading can be ignored for purposes of interpretation. Factor correlation matrix indeed revealed correlations between F1 with F6 (correlation coefficient=-0.443) and F3 with F6 (correlation coefficient=-0.292). Communalities for Item 18, Item 21 and Item 22 were 0.541, 0.746 and 0.517, respectively. This suggests the three variables/items contribute sufficient explanation to the variance. Furthermore, no substantial increase in Cronbach's alpha for any of the factors/subscales could have been achieved by eliminating more items. Hence, these three items were retained.

Factor solutions for 11, seven and six factors were each examined. The six-factor solution was preferred because: (i) the last factor extracted (F6) had an initial eigenvalue of 1.22, which was substantially higher than that of F7 (0.66), (ii) the Scree plot showed a clear inflexion point, with "leveling off" of eigenvalues on the Scree plot after six factors, and (iii) 67.19% of the variance was explained, the highest among the three-factor solutions.

Factors loadings for varimax rotation were displayed in a rotated factor matrix, while for oblimin rotation, the factor loadings appeared as a pattern matrix. Based on the final factor solution on the pattern matrix, the content of the items for each factor extracted was examined. The six factors were labeled as: F1=Comfort, F2=Interaction, F3=Aesthetics, F4=Autonomy, F5=Privacy, and F6=ICT facilities.

**Table 1:** Results of exploratory factor analysis for the 20 items of the revised LSQ (N=223)

Item No.	Item/variable	Rotated factor loadings						
		F1	F2	F3	F4	F5	F6	**C**
11	The lighting of the learning space.	**.689**						**.525**
12	The temperature of the learning space.	**.856**						**.680**
13	The comfort of the furniture.	**.845**						**.712**
14	The size of the working area.	**.639**						**.615**
22	Availability of water coolers/dispensers.	**-.419**						**.517**
23	Accessibility to washrooms.	**.698**						**.624**
6	I enjoy being with others.		**.813**					**.645**
7	I enjoy working with others.		**.883**					**.654**
8	I go to school for company too.		**.680**					**.413**
15	The use of colour in the building			**.761**				**.662**
16	The decoration of the learning space.			**.887**				**.726**
17	The presence of plants in the learning space.			**.678**				**.559**
9	I think it is important to decide for myself when I work on my study activities.				**.912**			**.760**
10	I think it is important to decide for myself where I work on my study activities.				**.943**			**.772**
4	I find it unpleasant when others can see what I do.					**.847**		**.567**
5	I find it unpleasant when others can hear what I say.					**.833**		**.569**
18	The presence of desktop computers.						**-.521**	**.541**
19	The presence of printing facilities.						**-.716**	**.550**
20	The presence of extension cables.						**-.664**	**.648**
21	The presence of charging stations.						**-.612**	**.746**
Eigenvalues		**6.19**	**2.64**	**2.21**	**1.62**	**1.36**	**1.22**	

Notes:  Extraction method: Principal axis factoring, Rotation method: Oblimin rotation with Kaiser normalisation factor loadings < 0.40 are not included; F1=Comfort, F2=Interaction, F3=Aesthetics, F4=Autonomy, F5=Privacy, F6=ICT facilities; C=Communalities

The factor loading matrix for this final solution is presented in Table 1.

### Reliability analysis

Reliability analysis returned an overall Cronbach's alpha of 0.845 for a sample of N=223. Reliability for each of the six factors were: Comfort (0.876), Interaction (0.833), Aesthetics (0.849), Autonomy (0.925), Privacy (0.841) and ICT facilities (0.800).

From the 40-item revised LSQ in five dimensions, a series of factor analysis resulted in the removal of 20 items and three dimensions. Hence, only findings related to the 20 items in the two dimensions that were retained (social dimension and physical dimension) are reported in this paper.

### Descriptive statistics

For the social dimension of the learning space, the 7-item dimension was extracted into three factors: "Interaction" (3 items), "Autonomy" (2 items), and "Privacy" (2 items).  The social dimension returned an overall mean of 3.88/5.00. The mean for "Autonomy" was the highest, while the mean for "Privacy" was the lowest (Table 2).

For the physical dimension of the learning space, the 13-item dimension was extracted into three factors: "Comfort" (6 items), "Aesthetics" (3 items), and "ICT facilities" (4 items). The physical dimension returned an overall mean of 4.21/5.00. The mean for "Comfort" was the highest, while the mean for "Aesthetics" was the lowest (Table 2).

## Discussion

The response rate of more than 70% was reasonably high, given the fact that online survey would have a recorded rate of around 30% to 40%.^30^

Barlett's test of sphericity is a statistical test for the presence of correlations among the variables. It provides statistical significance indicating the correlation matrix has sufficient correlations among at least some of the variables. The Kaiser-Meyer-Olkin (KMO) is a measure of sampling adequacy.^23^ A value close to 1 indicates that patterns of correlation are relatively compact, and so factor analysis should yield distinct and reliable factors.  Kaiser^27^ recommends values greater than 0.5 as acceptable.  In the final analysis, KMO recorded a value of 0.800, which is great.^23^ A significant Bartlett's test (p<0.001) indicates that correlations between the items are sufficiently large for factor analysis to be conducted.^23^  

Since no single criteria should be assumed to determine factor extraction,^29^ we had considered several criteria, including eigenvalues, Scree plot, and percent variance, explained when deciding on the number of factors to be retained after each factor analysis.

**Table 2:** Descriptive statistics related to social dimension and physical dimension of learning space (N=223)

Social dimension 7 items	Item Mean ± SD	Factor Mean ± SD
Item 4	3.48 ± 1.14	Privacy 3.51 ± 1.06
Item 5	3.53 ± 1.13	
Item 6	3.77 ± 1.04	Interaction 3.71 ± 0.93
Item 7	3.78 ± 1.00	
Item 8	3.58 ± 1.16	
Item 9	4.52 ± 0.72	Autonomy 4.51 ± 0.68
Item 10	4.49 ± 0.70	
Physical dimension 13 items	Item Mean ± SD	Factor Mean ± SD
Item 11	4.66 ± 0.65	Comfort 4.58 ± 0.09
Item 12	4.62 ± 0.69	
Item 13	4.51 ± 0.83	
Item 14	4.50 ± 0.84	
Item 22	4.47 ± 0.87	
Item 23	4.70 ± 0.67	
Item 15	3.71 ± 1.21	Aesthetics 3.69 ± 0.14
Item 16	3.59 ± 1.20	
Item 17	3.57 ± 1.26	
Item 18	3.48 ± 1.28	ICT facilities 4.09 ± 0.43
Item 19	4.10 ± 1.07	
Item 20	4.27 ± 0.99	
Item 21	4.49 ± 0.83	

The process of factor interpretation involves both objective and subjective judgement, and a wide range of issues need to be considered before a final solution is achieved.^21^ Hence, in our data analysis, several factor solutions with differing numbers of factors were examined before the factor structure was well defined. By examining a number of different factor structures derived from several trial solutions, we could compare and contrast before making the final decision on the most interpretable factor solution to represent the structure of the items/variables.^21^

In interpreting the factors from the initial analysis beginning with 40 items to the final factor solution with 20 items, the initial unrotated factor matrix was computed, containing the factor loadings for each variable and the factor. Factor rotation was found to simplify the factor structure to achieve simpler and theoretically more meaningful factor solutions. For the final stage, principal axis factoring of the remaining 20 items, using both varimax and oblimin rotations, was conducted, with six factors explaining 67.19% of the variance. An oblimin rotation provided the best-defined factor structure. According to Hair colleagues,^21^ few constructs in the real world are uncorrelated. Hence, oblique rotation is best suited to the goal of obtaining several theoretically meaningful factors/constructs that are correlated. Performing both rotational methods provides useful information on the underlying structure of the variables.

Table 1 shows all the factor loadings are at least 0.40. With sample size of 200 or more, factor loadings of 0.40 and higher will be considered significant for interpretative purposes.^21^ Although Item 8 had a communality of less than 0.5, it loaded fairly strongly on a single factor, "Interaction", with no significant cross-loadings. The percent of variance for each of the factor (not shown in Table 1) was recorded from EFA based on PAF with varimax rotation. A total of 67.19% of the variance was explained. A large proportion of the explained variance was associated with the first factor, "Comfort", which accounted for 18.67% of the variance. Other factors namely "Interaction", "Aesthetics", "Autonomy", "Privacy" and "ICT facilities" contributed 11.76%, 10.47%, 9.98%, 8.72% and 7.60% of the variance, respectively. A total variance was not available from EFA based on PAF with oblimin rotation. This is because when factors are correlated, sums of squared loadings cannot be added to obtain a total variance.

Reliability analysis for internal consistency reported an overall Cronbach's alpha of 0.845, indicating that the revised LSQ is a reliable instrument with good internal consistency.  With Cronbach's alpha ranging from 0.800 to 0.925, the six factors within the revised LSQ also have high internal consistencies.  Removal of Item 24 from the factor "Aesthetics" was deemed appropriate as the removal has caused the Cronbach's alpha for the factor to increase from 0.808 to 0.849.

This study provided evidence for the construct validity and internal consistency of the revised LSQ.  In terms of construct validity, a total of six factors in 20 items were extracted from the 40-item revised LSQ. The final factor solution for the revised LSQ explained 67.19% of the variance.  In terms of internal consistency, an overall Cronbach's alpha of 0.845 was recorded.

The validation data collected in this study was used to test the utility of the revised LSQ as a tool to investigate learning space preferences among Malaysian medical students. All the three items (Items 1, 2 and 3) in the dimension "Relevance of learning space" were extracted into the same factor but were excluded in the final analysis.  This finding was supported by Beckers and colleagues.^6^ For the social dimension of the learning space, all the seven items were retained, with the three aspects of the social dimension fitted into three factors: "Interaction", "Autonomy", and "Privacy". These findings concurred with the findings of Beckers and colleagues.^6^ However, for the physical dimension of the learning space, one item (Item 24) was removed.  Furthermore, the four aspects of physical dimension were reduced to three (Table 2).  These findings differed from findings by Beckers and colleagues.^6^ Of the three items in the aspect of "layout", two items (Item 22, Item 23) were extracted into the factor "Comfort" while one item (Item 24 – The transparency/openness of the learning space) was extracted into the factor "Aesthetics" but was excluded in the final analysis.  Hence, for the two dimensions (social and physical), only those items retained after the final EFA were included in the results and discussion of this paper.

An overall mean of 3.88/5.00 for the social dimension indicated that the medical students in this study did not perceive the social dimension of the learning space as very important.  However, the means of Items 10 and 9 (Autonomy) were relatively much higher compared to the other five items in the social dimension. Among the three factors in the social dimension, the mean for "Autonomy" was the highest (Table 2), suggesting that students perceived Autonomy as the most important factor in the social dimension.

An overall mean of 4.21/5.00 for the physical dimension indicated that the medical students in this study perceived the physical dimension of the learning space as more important compared to the social dimension.  Findings in Table 2 also suggest "Comfort" matters most to the students while "Aesthetics" is the least important.  All items in "Comfort" scored a mean of at least 4.50/5.00, indicating that students perceived "Comfort" as very important.  The fact that Item 23 scored the highest mean (4.70/5.00) among the 13 items suggested students considered accessibility to washrooms as very important.  This is good evidence that students responded truthfully as washrooms are basic amenities.  Item 18 (desktop computers) scored the lowest mean (3.48/5.00) among the 13 items.  With Wi-Fi access, the smartphone is an all-in-one device that students find useful and handy to use.

Many of the items in the two dimensions of "learning space preferences for individual study activities" and "learning space preferences for collaborative study activities" either had cross factor loadings or were extracted into factors that did not appear to be relevant constructs.  Hence, items in these two dimensions did not form part of the final factor solutions, and findings for these two dimensions were not reported in this paper. Drawing from the findings of Beckers and colleagues6 as well as this study, future studies can look into items in the dimensions of learning space preferences for both individual and collaborative study activities. Some of these items could be refined/rewritten for clarity.

Our study has its limitations. Firstly, only preclinical students from a single institution participated in the study.  Secondly, this study used non-random sampling methods which led to a lack of generalisation of the findings. We recommend the validity of the measure be further tested by extending the study to: (i) students in the clinical years in our institution, whose learning space during clinical postings involve hospital settings such as clinics and wards, and (ii) students from other public and private medical schools in Malaysia.

## Conclusions

This study established evidence for the construct validity and internal consistency of the revised questionnaire. Exploratory factor analysis with principal axis factoring identified a six-factor, 20-item solution, which explained 67.19% of the variance.  An overall Cronbach's alpha of 0.845 was reported, with Cronbach's alpha ranging from 0.800 to 0.925 for the six factors. Findings from this study provided insights on learning space preferences in medical school and suggested the revised questionnaire could be used as an instrument to investigate learning space preferences in Malaysian medical schools. In future research on learning space preferences, the revised questionnaire can be administered to a larger sample of students from other medical schools in Malaysia to test its robustness and to validate the revised questionnaire further.

### Conflict of Interest

The authors declare that they have no conflict of interest.
